# Evaluation of training, patient and practitioner perspectives on community-based monitoring of patients with stable age-related macular degeneration compared to hospital-based care: The FENETRE study report no. 1

**DOI:** 10.1111/opo.12836

**Published:** 2021-05-25

**Authors:** Simon Read, John G Lawrenson, Robert A Harper, Thomas Hanley, Konstantinos Balaskas, Heather Waterman

**Affiliations:** 1 School of Healthcare Sciences, Cardiff University, Cardiff, UK; 2 School of Health Sciences, City, University of London, London, UK; 3 Manchester Royal Eye Hospital, Manchester University NHS Foundation Trust, UK; 4 School of Health Sciences, Manchester University, Manchester, UK; 5 Moorfields Eye Hospital NHS Foundation Trust, London, UK; 6 Formerly, School of Healthcare Sciences, Cardiff University, Cardiff, UK

**Keywords:** accreditation, clinical competence, optometry, primary health care, secondary care, wet macular degeneration

## Abstract

**Purpose:**

Describe the development, delivery, acceptability and evaluation of a modular training programme for community-based, non-medical practitioners monitoring patients with quiescent neovascular age related macular degeneration (QnAMD). Also, report on a qualitative process evaluation conducted during the pilot phase of a randomised control trial (the FENETRE Study) exploring patient and practitioner acceptability of community-based QnAMD care relative to hospital-based care.

**Methods:**

Learning outcomes from The College of Optometrists’ Medical Retina higher qualifications and the Royal College of Ophthalmologists’ Common Clinical Competency Framework were used to develop a competency framework for QnAMD care. Training was delivered online, comprising six asynchronous lectures followed by two synchronous case-based discussion webinars, with an accredited assessment of 24 case vignettes. An anonymous evaluation survey was conducted with the first two FENETRE cohorts (*n* = 38). Separately, we undertook a qualitative process evaluation, sampling purposively in four hospitals and five community-based practices, interviewing nine patients and eight practitioners.

**Results:**

Survey responses (*n* = 26) showed community optometrists were very satisfied (*n* = 12; 46%) or satisfied (*n* = 14; 54%) with the training; feedback reflected by qualitative process evaluation data. Overall, optometrists also felt either confident (*n* = 15; 58%) or very confident (*n* = 8; 31%) in conducting AMD monitoring appointments following training, a finding also corroborated by interview data from optometrists participating in the initial pilot phase roll-out. Optometrists identified patient convenience and alleviating pressures in hospital care as the primary reasons for acceptability of community pathways. Data from patients entering community practices suggested they largely found this at least as safe and convenient as hospital care, although some patients randomised to hospital care perceived that as safer.

**Conclusion:**

This pilot study has shown the development and implementation of a collaborative community monitoring model is feasible, with satisfaction from community optometrists for training and accreditation, and broad acceptance for the pathway by both patients and practitioners.

**Supplementary Information:**

The online version of this article (doi:10.1111/opo.12836) contains supplementary material, which is available to authorized users.

## Introduction

Age related macular degeneration (AMD) is the leading cause of ocular morbidity in high income countries,^1^ with the disease burden predicted to rise by ~60% in the next 20 years.^2^ Analysis of recently available data on certifiable visual loss in England and Wales indicated that AMD was responsible for approximately 50% of new certifications for severe sight impairment.^3^ Although non-neovascular (dry) AMD is by far the more prevalent, the neovascular (wet) form (nAMD) of the disease accounts for the vast majority of cases of severe visual loss (worse than logMAR 1.0).^4^

The accepted standard of care for patients with nAMD is intravitreal anti-vascular endothelial growth factor (VEGF) treatment.^5^ These drugs render nAMD quiescent through regression of choroidal neovascular membranes, with a corresponding reduction in fluid leakage into the retina.^6^ Since there is a high risk of reactivation, regular clinic visits are required to detect reactivation and identify the need for further treatment. Monitoring typically involves measuring visual acuity, a retinal examination and optical coherence tomography (OCT) imaging, although some services extended their use of virtual models and/or adopted other changes during the coronavirus pandemic.

The need for regular monitoring of quiescent nAMD (QnAMD) places considerable demands on Hospital Eye Services (HES) in terms of space and staffing with innovative care pathways developed to manage capacity issues in hospital services and maintain high quality provision.^7^ Although optometrist-led QnAMD clinics are relatively common in the hospital setting, collaborative models of care using accredited community optometrists have so far received relatively little attention. In 2016, the Effectiveness of Community vs HES (ECHOES) trial demonstrated that community optometrists were equivalent to ophthalmologists in making retreatment decisions in patients with QnAMD based on clinical vignettes. However, qualitative research conducted alongside the main study identified a number of potential barriers, including ophthalmologists’ perceptions of optometrists’ competence; the need for clinical training and the ability of professions to work collaboratively.^8^

This paper reports on the development and pilot phases of a randomised control trial (the FENETRE Study) exploring safety and acceptability of community-based monitoring of patients with QnAMD, relative to hospital-based clinics. For clarity, when referring to community care in this context, we are referring to the management of secondary care QnAMD cases in primary care settings by community optometrists. We describe the development, delivery and evaluation of a modular training programme for community optometrists and present results of a qualitative process evaluation conducted during the pilot phase of the study, prior to the first UK COVID-19 lockdown (23 March 2020), to evaluate patient and practitioner acceptability of community care.

## Methods

The FENETRE Study was designed as a prospective randomised multi-site clinical trial recruiting QnAMD patients from hospital sites.^9^ Once recruited, patients are randomised into either community or hospital-based study cohorts, with the aim they would receive QnAMD care in that setting over 12 monthly follow-ups (subsequently extended to 8 weekly reviews during COVID-19) before returning to hospital-based care. Hospital care incorporated eye services varying between optometrist/ophthalmologist-led face-to-face and virtual clinics where ophthalmic data were collected, mainly by technician staff, and later reviewed by more senior optometrists or ophthalmologists. This article focuses on two objectives within the trial’s pilot phase:

1. To develop a scalable training programme for QnAMD care delivery for community optometrists; and
2. To assess patient and practitioner acceptability of community QnAMD care pathways.

The Quality-Assured Follow-up of quiEscent Neovascular ageE-relaTed macular dEgeneration by non-medical practitioners (FENETRE) trial received a favourable ethics opinion (REC reference 18/LO/2111), including the qualitative and quantitative work described herein. Qualitative data are reported in line with the consolidated criteria for reporting qualitative research (COREQ). The COREQ checklist is included as supporting information (*Appendix* S1).

### Development and evaluation of the FENETRE training programme

#### Competency framework development

The College of Optometrists (The College) has developed a series of higher qualifications in Medical Retina (Certificate, Higher Certificate and Diploma) incorporating diabetic retinopathy screening and referral and treatment pathways for AMD.^10^ In parallel, the Royal College of Ophthalmologists (RCOphth) produced the Ophthalmic Common Clinical Competency Framework (CCCF), providing standards and guidance for the knowledge and skills required for non-medical eye healthcare professionals, working in a hospital setting within a multidisciplinary team, to deliver patient care.^11^ The CCCF for Medical Retina incorporates three levels:

- Level 1. To enable participation in screening, under supervision, of medical retina patients and participate in monitoring low risk patients with established diagnoses in protocol driven treatment clinics;
- Level 2. To enable participation in triage and assessment of new patients and perform assessment, management and monitoring under specific protocols;
- Level 3. To enable participation in hospital based medical retina patient care, managing and discharging patients under the care of a consultant ophthalmologist.

The learning outcomes from each of the three College Medical Retina higher qualifications and the RCOphth CCCF were used to develop a bespoke competency framework to deliver QnAMD care within the FENTERE study. The framework covered Background Knowledge; History Taking; Assessment; Diagnosis; Management and Communication/Governance. A number of study specific competencies were also included. The competency framework is available in *Appendix* S2.

#### Programme for training and accreditation

Community optometrists were recruited to the study with the assistance of local networks including Local Optical Committees and practitioners known to hospital eye services, incorporating both larger chain and smaller independent practices, as well as a range of experience levels. To be eligible for participation in FENETRE, participants needed to meet the following criteria:

- be registered with the General Optical Council for at least 2 years, and
- be practising within the General Optical Services (GOS).

The training programme was delivered entirely online using Moodle, the online learning platform used by City, University of London (moodle.org) and consisted of six asynchronous lectures:

- Epidemiology, classification and pathogenesis of AMD;
- Clinical presentation of AMD;
- Optical coherence tomography in AMD diagnosis;
- Pharmacological management of AMD;
- Monitoring of QnAMD and criteria for retreatment;
- AMD case scenarios.

In addition, participants were invited to attend two synchronous case discussion webinars, with an emphasis on differentiating active from QnAMD and making re-treatment decisions. A final webinar covered the study protocol for FENETRE. Webinars were recorded and made available as additional online resources. Participants were allowed to progress through the training at their own pace but typically the material was covered over a 12-week period, corresponding to approximately 4 h per week.

On completion of the training programme, participants undertook an accreditation assessment similar to that used in the ECHOES trial^8^ and comprising 24 case vignettes that were representative of the clinical decision-making process required within the FENETRE trial. Each vignette incorporated retinal images (colour fundus photographs and OCT scans) at two time points (baseline and follow up), with accompanying clinical data (gender, age, smoking status and best corrected visual acuity (BCVA)). The ‘baseline’ corresponded to a visit where nAMD was deemed to be quiescent. For the follow up visit participants were asked to: 1. Identify the clinical features present; 2. Determine the clinical classification of patients (quiescent or re-activated) and 3. Make a patient management decision (either continue monitoring in the community or referral to hospital eye clinic). The passing score for the assessment was based on assigning ‘correct’ activity status and management decisions for at least 75% (18 of 24) of the vignettes. This pass rate was aligned with the similar validated case-based approach and accreditation level deployed in the ECHOES study.^8^

#### Evaluation of the training programme

An 18-item evaluation survey was conducted using Qualtrics software (qualtrics.com). A link to the questionnaire was emailed to participants in the first two FENETRE cohorts (*N* = 38) once accreditation was complete. The survey was divided into sections covering benefits of online lectures and webinars, training delivery, time taken to complete training elements, level of difficulty of final assessment and confidence that training had prepared participants to see study patients. For this article, we primarily report on survey items relevant to the qualitative process evaluation as opposed to those on the delivery of the training itself. Respondents were assured that responses would be anonymous so as to reduce any potential bias.

### Qualitative process evaluation

The pilot phase qualitative process evaluation addressed the objective of assessing initial patient and practitioner acceptability of the care pathways through two interlinked research aims:

i. Determine how the implementation of the community based QnAMD clinics can be improved for the main study;
ii. Identify corresponding contextual factors that underpin how and why the clinics work.

Data were collected between December 2019 and March 2020, prior to the first UK COVID-19 lockdown, by a researcher independent of the development and delivery of the training programme (SR). All participants were also reassured that the interviews and observations would be confidential to ensure freedom of expression and reduce the risk of bias. Of six hospital sites active during the pilot study, four were visited for observations and interviews, while of 13 active community practices, five participated in the research, mirroring or exceeding the recruitment targets of four sites in each setting intended for the pilot phase.^9^ Sampling was performed purposively with sites being selected based on geographical diversity and size of practice, as well as being restricted to those sites where active FENETRE appointments were taking place.

#### Observational field notes

Observational field notes were taken in-clinic before, during and after clinical consultations and documented any practical, organisational, professional or behavioural issues in implementation that would not typically arise during interviews. Additionally, field notes included observed variations in practice between and within hospital or community practices. Observations were collected over a total of nine visits, comprising ~10 h.

#### Patient interviews

Semi-structured interviews were conducted with patients attending either hospital or community QnAMD appointments. Nine patients were interviewed, five having received community care with four randomised into HES. Open-ended schedules were developed to investigate how patients access clinics and their views on being seen in either setting, what changes they would make to clinic organisation, whether staffing and frequency of appointments were adequate and their views of the care received (see *Appendix* S3). Interviews were audio-recorded and lasted 15–35 min.

#### Community optometrist interviews

Five community optometrists were invited to participate in semi-structured interviews. These were conducted after observations of consultations, with this data contributing towards the interview schedule. Schedules also explored participants’ views on FENETRE training and any elements they would change, the extent to which their practice was reorganised to accommodate FENETRE appointments, and any impacts on service delivery to other patients (*Appendix* S3). Additionally, optometrists were asked whether they felt the FENETRE pathway would achieve its aim of managing QnAMD in community care, taking into account patient safety, outcomes, experience and access to care. Interviews lasted 15–40 min.

#### Hospital-based practitioner interviews

Interviews were also sought with optometrists and ophthalmologists involved in delivering QnAMD care in HES. Again, these were conducted after observation field notes of a standard patient appointment had been taken. Three semi-structured interviews were carried out; two with HES optometrists and one with an ophthalmologist. Schedules covered practitioner perspectives of QnAMD service delivery in their setting, as well as views on the FENETRE pathway and potential barriers to its implementation (*Appendix* S3). Interviews were audio-recorded and lasted 15–40 min.

### Data analysis

Survey data were descriptively analysed using Qualtrics in-built software, with the research team extracting results as reported.^12^ Qualitative data were organised in Nvivo (QSR International, qsrinternational.com)^13^ with a deductive thematic framework analysis approach adopted to ensure systematic rigour.^14^ Regular meetings were held between the research team to discuss data interpretation, as well as revise and adapt the final thematic framework (see *Appendix* S4).

## Results

We discuss our findings initially in terms of development of the training programme and how this was perceived by those enrolled in it. For these purposes, responses from the Qualtrics survey are reported alongside qualitative feedback gathered from the pilot process evaluation. Thereafter, we discuss the acceptability of FENETRE appointments initially from the patients’ perspectives, and then from practitioners in community and hospital settings.

### Training programme feedback

#### Overall satisfaction

The Qualtrics survey was issued to 38 community optometrists actively engaged in the FENETRE training and received 26 responses; a response rate of 68%. Survey responses outlined broad overall satisfaction with online training. All those providing answers stated they were either very satisfied (*n* = 12; 46%) or satisfied (*n* = 14; 54%). This broad positive feedback was reflected in the qualitative data:

*‘I thought there might be elements of it that…you don’t really need to know or that aren’t very applicable, but it was very good*.*'*

*‘The training…was very thorough from the point of view of taking you right back to basics on retina, normal structure, what can go wrong, treatment of people with wet macular degeneration, all sorts of interesting things on anatomy and treatment and a lot about profiles of who gets it.' LIH-001 TUH-004*

Participants highlighted the thoroughness of the programme overall, and provided several examples where online learning surpassed their expectations. Though tacit familiarity with learning materials varied between participants, feedback was positive from those with both high and low levels of previous experience of medical retina.

#### Usefulness of individual topics

Survey data suggested all six learning topics of the training were felt to be predominantly either very beneficial or beneficial (*Table* 1).

**Table 1:** Survey responses on perceived benefit of Follow-up of quiEscent Neovascular ageE-relaTed macular dEgeneration by non-medical practitioners (FENETRE) learning topics

Topic	Very Unhelpful (*n*; %)	Slightly Unhelpful (*n*; %)	Beneficial (*n*; %)	Very Beneficial (*n*; %)	Total (*n*; %)
1: Epidemiology, classification and pathogenesis of AMD	1; 4%*	1; 4%	17; 65%	7; 27%	26; 100%
2: Clinical presentation of AMD	1; 4%*	0; 0%	15; 58%	10; 38%	26; 100%
3: Optical coherence tomography in AMD diagnosis	1; 4%*	0; 0%	11; 42%	14; 54%	26; 100%
4: Pharmacological management of AMD	1; 4%*	2; 8%	17; 65%	6; 23%	26; 100%
5: Monitoring of QnAMD and criteria for retreatment	1; 4%*	0; 0%	12; 46%	13; 50%	26; 100%
6. Case scenarios	1; 4%*	0; 0%	9; 35%	16; 61%	26; 100%

AMD, age related macular degeneration; QnAMD, quiescent neovascular age related macular degeneration.

*Suspected that participant entered incorrect values – was otherwise satisfied with training.

The majority of participants saw benefit in each of the learning topics with the lowest rated, pharmacological management of AMD, seeing 88% of respondents rate it as either beneficial or very beneficial. The core procedures of assessing optical coherence tomography (*n* = 14; 54%), monitoring QnAMD and criteria for retreatment (*n* = 13; 50%) and related case scenarios (*n* = 16; 61%) were perceived as most beneficial overall potentially reflecting their interlinkage with the primary task of differentiating between active and quiescent nAMD. Again, this was demonstrable in feedback received through qualitative interviewing. While overall satisfaction was reportedly high, some participants expressed the desire for more learning opportunities in certain areas:

*‘Looking at more OCT scans with somebody talking through ‘this is this, that is that, we’re not referring that one because of this’ more of that practice maybe might have been good.' TUH-004*

Thus, while participants reported topics on the interpretation of scans (unit 3) and associated case scenarios (unit 6) as more beneficial, this reflects that participants were broadly satisfied with them but may also benefit from increased content.

#### Training delivery

Duration of online lectures for each learning topic varied from 13 min (Topic 2) to 70 min (Topic 3) with the total duration of all online lectures ~4.5 h. Additionally, some survey respondents (*n* = 17; 65%) stated they did additional study beyond the provided content. Of those doing so, additional study varied in length from up to 30 min (*n* = 2; 12%), 30 min to 1 h (*n* = 2; 12%), 1–2 h (*n* = 3; 18%), 2–4 h (*n* = 6; 35%) or over 4 h (*n* = 4; 24%), corroborating findings on the potential to include more content on key learning topics. Resources used for such study were stated as online research, peer to peer discussion, further reading/note-taking and studying OCT images.

Respondents engaging with the online training webinars rated them collectively as either very beneficial (*n* = 25; 53%) or beneficial (*n* = 22; 47%). Further data were also collected on elements of functionality within webinars, such as interactive chat enabling communication between audience and presenters and relatedly the difference between live and recorded presentations. While some participants (*n* = 3; 14%) felt they would rather be able to communicate online with their voices as opposed to text chat, the majority (*n* = 18; 86%) did not feel that this limited participation. Similarly, it was noted that a greater proportion of participants felt it either important (*n* = 10; 48%) or very important (*n* = 7; 33%) they were able to communicate with presenters in real-time, preferring live over recorded webinars. When questioned on mode of delivery during qualitative interviews, all participants stated they found webinars and general online delivery satisfactory, even for those with a predisposition to disengage with online learning:

*‘I think some people really enjoy online learning, I generally don’t, because of the fact that I tend to, I lose concentration and I start looking or doing something else…whereas this was really good.' DAH-001*

#### Assessment and preparedness for FENETRE

The majority of community optometrists stated they found the practice assessment either beneficial (*n* = 13; 50%) or very beneficial (*n* = 12; 46%). Furthermore, most respondents found the final assessment difficult (*n* = 16; 62%) as opposed to very difficult (*n* = 1; 4%), slightly easy (*n* = 8; 31%) or very easy (*n* = 1; 4%). These findings correlate with qualitative interview data where, in spite of a range of experience in relation to QnAMD, there was an acknowledgement that training and assessment was suitably challenging: *‘My background’s not really in medical retina, and so I found it quite challenging, but as I say, at our level, it was definitely doable'* DAH-001; *‘So the preparation on passing the exam and looking at OCT scans and things I thought was good'* TUH-004.

Regarding preparedness to host FENETRE appointments post-training, the majority of survey participants stated they felt either confident (*n* = 15; 58%) or very confident (*n* = 8; 31%), corroborated by interview data where participants expressed understanding of the clinical procedures and interpretation of optical coherence tomography. One caveat, however, came in the translation of training into the applied environment. Though not widely reported as an issue, several participants mentioned they were performing FENETRE appointments for only the first or second time during interview:

*‘The bit that wasn’t absolutely clear was how it was actually going to happen with real patients in this real practice…but it is a little bit of a step of faith, if you like, "well, before I’ve used all of this, I don’t really know how it’s all going to work" TUH-004*.

### Patient acceptability

For patients randomised into community care, data suggested they largely found this arrangement at least as acceptable as hospital appointments. The following interview extracts demonstrate broad patient satisfaction post-appointment: *‘I think it went very well indeed. They listened to me'* LI-002; *‘I’m more than happy with everything…it’s fantastic'* DA-008; *‘I’m quite happy to come to community care'* TU-009. Nevertheless, patient acceptability was often nuanced with regard to factors such as physical environment of practices, timeliness of attending appointments, concerns over capabilities of community practitioners and the experience of patient-practitioner interactions.

#### Physical environment issues

Most patients randomised into community care practices responded favourably to these new arrangements, noting that spaces were generally quieter with fewer patients. However, one participant randomised into a larger, city centre practice noted elements of the physical environment that impacted their satisfaction:

*‘I felt the environment of the shop was actually not good, and it’s very noisy and it’s very crowded and they had a very grubby carpet, and if you’re going in hospital, that felt quite different.' RO-003*

Observations and interviews broadly demonstrated community care practices to be quieter, although it was noted that the physical layout of commercial practices notably contrasted to other smaller independents. A further issue associated with physical layout in practices was key equipment being situated on the first floor in two sites. One practice offered an external elevator for patients, though the other could not due to it being a listed building. While patients observed in each practice were able to navigate stairs, both mentioned this as an issue:

*‘The stairs are actually quite steep, they don’t have a lift (elevator), she said ‘They couldn’t have a lift put in because it’s a listed building’…but I recently had an operation on my knee.' RO-003*

*‘I thought the stairs were a bit steep and I did wonder how people who are, perhaps not as agile as myself, would get up there.' TU-011*

The hospital setting was characterised as busy and crowded by most patients. While most hospital sites operated virtual clinics, offering greater timeliness due to ophthalmic data being reviewed after the patient has left the appointment, one site required patients to see an optometrist in clinic, prompting significant delays: ‘ROH-001 stated that the clinic was extremely busy most of the time with patients commonly waiting 90 to 120 min for their appointment to complete’ 200120, ROHosp. Interestingly, patients generally associated the hospital environment with busyness regardless of whether the clinic was virtual or not:

*‘I have the scan, then I go back and sit and wait, and then eventually and there’s a lot of people there, I’m called then to see the Specialist.' DA-008*

#### Transport and timeliness of appointments

The timeliness of appointments appeared to influence patient experience. Yet, while community care appointments were generally longer than hospital-based virtual clinic slots, this was not perceived unfavourably. Additionally, time taken to reach appointments was also an important consideration for patients. Patients offered similar viewpoints on the ease of reaching community practice appointments as opposed to attending a centralised eye hospital:

*‘It’s easy for me to get there…but I can imagine if you lived further afield, you would travel for an hour to go to a five minute review session, that might feel a bit, the proportions wouldn’t be right then.' DA-002 (Hospital)*

*‘Woke up this morning, obviously got ready, my husband…came with me, because I knew I wouldn’t be able to drive home, so I just parked round the corner, I only live less than five minutes away.’ DA-008 (Community)*

#### Concerns for community capability

For those randomised into community care, it was felt that the clinical procedures carried out were broadly equivalent to those in hospital and performed to the same levels of patient safety: *‘They did the same sorts of tests that I would normally have done at the eye hospital. But the ones I had done there (in community) were far more intense'* LI-002; *‘He explained to me that they were people that they’d trained, so I knew by them saying that to me, that I would be getting that ongoing care'* DA-008.

Some patients randomised to remain in hospital care expressed satisfaction with the care received due to perceptions of it being safer than community care:

*‘Can they pick up on everything that they can at the eye hospital? Okay, you are cutting down on my time, half an hour instead of two hours. But I don’t mind sitting there for 2 hours if they know what they are doing.' RO-001*

Indeed, this concern was voiced by several participants still receiving hospital eye service care with wider anxieties related to their sight and the management of their condition taking priority over issues such as travel or convenience:

*‘As good as someone in the community might be, for my little brain this is where I would like to be and I feel if anything untoward is noticed at any time, I’m where I need to be.' TU-012*

#### Patient-practitioner interactions

Patients randomised to community care appeared to value interactions often embedded into their appointments, particularly those relating to their health outcomes. Observations and interviews in the virtual clinic environment of the hospital setting highlighted that limited outcome information was provided to patients. Patients were primarily reliant on post-appointment letters outlining their follow-up, often providing limited clinical detail. When compared to the patient-practitioner interactions afforded in community care, there were greater opportunities for discussion:

*‘DA-008 asks if this information is being sent over to the eye hospital. DAH-001 explains that it all will be and then begins talking the patient through the appointment outcomes…suggesting that the AMD remains stable.' 200221; DAComm*

*‘TUH-005 begins talking the patient through the images and what they mean. Patient appears to value this additional information.' 200206; TUComm*

### Community optometrist acceptability

For independent community care practices FENETRE appointments were felt to offer new opportunities for learning and development, as well as a broader range of work activity:

*‘I think nine to five, Monday to Friday, every routine eye examination, can be a little bit monotonous and so from a clinical perspective, I think it’s that bit more challenging, it’s that bit more variation.' DAH-001*

*‘It’s interesting, it’s a learning curve, and it’s something that I’ve not done before, but definitely a skill I want to improve.' LIH-001*

*‘It gives me something else to learn. And with all the advancing, not just technology, but treatment and protocols and all that, I think it’s just something that I’ve always been interested in.' TUH-004*

Most optometrists working in FENETRE community practices expressed the benefits of learning at an individual level, often relating this to the training programme. However, data highlighted concerns that response to FENETRE appointments may differ based on size of the practice. For smaller, independent practices, optometrists identified the benefits of incorporating additional work:

*‘Having kind of expended enhanced schemes like this, is actually very important from a financial and business perspective for them.' DAH-001*

In one practice it was also noted that a patient arranged an additional appointment to manage other sight issues. As such, there was potential for further appointments to be made, where appropriate, which may benefit smaller practices: *‘There's probably a commercial benefit to opticians in that they establish a relationship with the patient'* ROH-004. However, concerns were expressed around whether financial incentives within FENETRE would be adequate for larger commercial practices:

*‘My slight worry is that some of the more commercially minded optometrists, may not actually take it on board, because it’s not going to enable them, they’ll lose money if they do it.' LIH-004*

All community optometrists reported feeling comfortable performing required clinical procedures: *‘I think it’s exactly as it would be done in a hospital'* LIH-001; *‘In terms of how the consultation went with the patient, it went absolutely fine, I think it went well'* DAH-001. Many participants were already trained in routine care procedures such as visual acuity, slit lamp examination, dilated fundus examination and OCT scanning. Furthermore, the cases reviewed in community care clinics were generally not perceived to be complex by the optometrists overseeing them. Nevertheless, given that observations were performed during optometrists’ first or second FENETRE appointments, issues with familiarisation were noted:

*‘Because it was the first patient we didn’t really know what timing to give it, so we had booked it in for a time and gave breathing space. But had we actually just booked it in for the time we thought might be suitable, I would have been really, really, really rushed.' LIH-001*

*‘So it’s just the second patient episode that I’ve done so it’s still kind of a little bit of a feeling of getting used to what needs to be done in that slot because we are so used to doing things in our routine and we have to think outside of that.' TUH-004*

The FENETRE training reportedly assisted with much of the sense-making clinical work but when implementing the intervention, those quoted above imply elements of uncertainty on the composition of appointments and wider processes. Beyond this, no reports of negative feedback or poor communication and collaboration from HES staff were made, with smooth working reported by all participants.

### Hospital-based practitioner acceptability

Hospital-based optometrists and ophthalmologists were predominantly interviewed around their acceptability of monitoring QnAMD within the community setting. Most identified numerous reasons as to why it was more acceptable than current arrangements, with no participants highlighting issues of competence or poor relations with ophthalmologists as found in the ECHOES study.^8^ Many related positive perceptions to patient acceptability, suggesting community care may be less busy and hectic to navigate, with appointments potentially being timelier and offering greater opportunity for communication:

*‘We’re struggling at the moment to get people back at four or five weeks, because we haven’t got the appointment space…so therefore it would be much better from a patient’s point of view… if they could be seen at the local optometrist.' ROH-001*

While patient acceptability was mentioned by all hospital staff, this was generally interrelated with increasing capacity within hospital eye services: *‘The primary reason why this is such a good idea is that it increases capacity without causing any additional burden to space and staff'* LIH-004. Such perspectives mirror the wider study aims of FENETRE and demonstrate the perceived value from both community and hospital-based practitioners.

## Discussion

This initial study has demonstrated the feasibility and acceptability of a competency-based training model providing the knowledge and skills required for monitoring of QnAMD in community care. The major strength of the training programme was its online delivery, allowing participants to progress at their own pace, accessing the learning material at a time convenient to them. This feature is particularly important for busy practitioners, who would otherwise need to leave their practices to attend didactic training. The training was underpinned by a competency framework, developed from the learning outcomes of the CoO higher qualifications in medical retina and the RCOphth medical retina CCCF. In the future, this commonality would allow practitioners with relevant professional qualifications to gain exemption through recognition of prior learning. The accreditation process for FENETRE was based on an online clinical vignette-based method, previously validated as part of the ECHOES trial.^8^

Recruitment of community optometrists relied on self-selection due to the requirement of undertaking an enhanced role within their organisation, as has been outlined in previous studies.^15^ While efforts were made to offer as broad a presentation as possible through engagement with local networks and Local Optical Committees, the sample of optometrists may not be representative of the wider population as a whole. That said, should the service be rolled out in the real-world, similar processes of self-selection would also be anticipated.

Sample sizes for the qualitative process evaluation were limited due to this being the pilot phase of a wider randomised control trial, with themes and issues emergent from such work to be further explored into the main study. Additionally, recruitment was limited to the hospital and community practices engaged with the FENETRE study at the time of the pilot. Nevertheless, the semi-structured interviews offered rich data on the experiences of practitioners, highlighting broad satisfaction in the handling of community FENETRE appointments and only minor familiarisation issues. This suggested FENETRE training fulfilled its purpose in terms of practitioner confidence in medical retinal interpretation. Similarly, hospital-based practitioners perceived the principles of FENETRE favourably both from the perspective of patient experience and alleviation of NHS capacity issues. Interestingly, no data collected during the pilot indicated concerns from hospital practitioners relating to the capabilities of community optometrists, deviating from ECHOES study findings.^8^ There is potential that this may emerge more prominently as a theme within the main trial phase as the study expands to other sites. Finally, data suggested there may be significant nuance in how FENETRE is perceived by community care practices based on their size, with larger practices potentially seeing less benefit than smaller independents. Such concerns highlight variability in how practices may respond to FENETRE pathways in the long-term. For the independent practices, the FENETRE pathway may prove to be an important source of additional income and professional development, whereas there are concerns that the larger commercial sector structures may perceive these as less viable.

Patients randomised into community care practices reflected positively on their care, highlighting such factors as enhanced patient-practitioner interactions, as well as potential for increased convenience. Some concerns for capability in community care were expressed by those randomised into hospital care, albeit having had no experience of it. As these concerns were largely around safe monitoring of their QnAMD, it suggests there may be need for further patient reassurance that community care will equate to that of hospital-based care. Furthermore, patients generally reflected favourably on the convenience of visiting community optometrists, with significantly reduced journey times in several instances. That said, some locales offered less of a geographical spread for patients, limiting perceived benefits. As the number of practices expands into the main study, it is anticipated that this problem may become less noticeable. Relatedly, some patients highlighted that community practices differed from the hospital setting with regard to physical access. Some practices were unable to offer lifts to upper floors, which may be impractical for those with mobility issues. Again, it is anticipated that, with a wider range of community optometrists integrated into the main study, sites offering greater physical access can be selected at the point of randomisation.

In conclusion, while the pilot phase demonstrated some minor challenges for community practices, the feedback from both the training programme and the qualitative process evaluation demonstrate the potential of the FENETRE pathway. The benefits of alleviating pressure on hospital-based eye services were broadly understood by all participants, with procedures and background knowledge felt to be assured either through pre-existing skill sets or through the accredited training. The limitations outlined above will be monitored into the main study, with the anticipation that the expansion of new sites will contribute further findings that benefit the future uptake of the pathway, which appears to be needed now more than ever in a challenging environment for ophthalmic healthcare.

## Supplementary Information

**Appendix S1**. Consolidated criteria for reporting qualitative studies (COREQ): 32-item checklist.

**Appendix S2**. FENETRE training competency framework.

**Appendix S3**. Qualitative interview schedules.

**Appendix S4**. Qualitative analytical framework.
