# Supplementary material for: Evaluation of training, patient and practitioner perspectives on community-based monitoring of patients with stable age-related macular degeneration compared to hospital-based care: The FENETRE study report no. 1
Source: Ophthalmic Physiol Opt. 2021 May 25;41(4):864–73. doi: 10.1111/opo.12836 (PMC12852252; doi:10.1111/opo.12836)
Supplement: Supplementary file 2 — Appendix S2. FENETRE training competency framework. [file 44402_2021_4104021_MOESM2_ESM.docx]

# *The FENETRE Study Report No. 1*

# Supplementary Appendix S2 – FENETRE Training Competency Framework

This document provides a framework that defines the knowledge, skills and attributes required for non-medical eye healthcare professionals to manage nAMD in the context of the FENETRE study. The competencies are derived from existing medical retina frameworks that were developed to allow non-medical eye healthcare professionals to participate in medical retina pathways in primary or secondary care.

- The Common Clinical Competency Framework for Non-medical Ophthalmic Healthcare Professionals in Secondary Care: Medical Retina. Royal College of Ophthalmologists November 2016.

<https://www.rcophth.ac.uk/wp-content/uploads/2017/01/CCCF-Medical-Retina.pdf> (accessed 10.10.18)

- Higher Qualifications in Medical Retina. College of Optometrists

<https://www.college-optometrists.org/cpd-and-cet/training-and-qualifications/higher-qualifications/courses-and-providers/higher-qualifications-in-medical-retina.html>

(Accessed 10.10.18)

This competency framework will be used to develop the training programme to accredit community optometrists to participate in the Fenetre study.

1. **Background knowledge**
   1. A detailed knowledge of the anatomy, physiology and pathophysiology of the retina, with emphasis on the macula
   2. An understanding of the risk factors and differential diagnosis of AMD
   3. An understanding of treatments of AMD including the patient’s response to treatment
   4. An awareness of the rapidly evolving nature of AMD treatments including pertinent treatment trials
   5. An awareness of clinical trials which may influence current and future practice
   6. An understanding of current guidelines for management of AMD
2. **History-taking**
   1. An ability to re-evaluate a clinical history, including an ability to elicit and understand the relevance of any significant symptoms relevant to change in clinical status
3. **Assessment**
   1. An ability to undertake a detailed slit lamp examination including Volk lens indirect ophthalmoscopy
   2. An ability to interpret OCT images and fundus photographs for AMD with appropriate patient management
   3. An ability to use and interpret OCT imaging software and fundus photography to review data and make accurate diagnoses
4. **Diagnosis**
   1. An ability to diagnose nAMD with a provisional recommendation for treatment
   2. An ability to detect retinal thickening and recognise its importance in determining treatment
   3. An ability to use and interpret OCT imaging software and fundus photographs to review data and make accurate diagnoses
5. **Management**
   1. An ability to make re-treat decisions for nAMD according to local protocols in a consultant ophthalmologist-led pathway, including the ability to determine when further investigations are required in the event of atypical or sub-optimal responses to treatment
   2. An ability to diagnose nAMD with a provisional recommendation for treatment
   3. An ability to differentially diagnose retinal and macular conditions and manage or refer as appropriate
6. **Communication/governance**
   1. An ability to communicate with patients about their diagnosis, treatment plans and outcomes
   2. An ability to communicate with colleagues within a multidisciplinary setting
   3. An ability to work within the clinical governance framework of the ophthalmic unit
   4. An ability to manage patients’ fears and concerns
7. **FENETRE study specific**
   1. An understanding of the governance of the NIHR project Fenetre
   2. An understanding of the Fenerte study protocols and the role of participant community optometrists
   3. An ability to deal with patients’ study specific queries and refer to the PI/CI as required
