# Supplementary material for: Evaluation of training, patient and practitioner perspectives on community-based monitoring of patients with stable age-related macular degeneration compared to hospital-based care: The FENETRE study report no. 1
Source: Ophthalmic Physiol Opt. 2021 May 25;41(4):864–73. doi: 10.1111/opo.12836 (PMC12852252; doi:10.1111/opo.12836)
Supplement: Supplementary file 3 — Appendix S3. Qualitative interview schedules. [file 44402_2021_4104021_MOESM3_ESM.docx]

# *The FENETRE Study Report No. 1*

# Supplementary Appendix S3 – Qualitative Interview Schedules

**Appendix C1: Patient Semi-structured Interview Schedule**

This topic guide should be used as reference prior to conducting qualitative interviews with patients as part of the FENETRE Study’s Process Evaluation in the internal pilot and main study. The precise questions used will vary according to what was discussed and carried out during the patient-HCP consultation, with some questions referring directly to events within it. Examples of these types of question are included below with XXXXX marking content to be drawn from the observations taking place beforehand. The interviews will be semi-structured and will explore the perspectives of patients involved in consultations with nAMD staff and their understanding of these interactions.

**General Appointment Issues**

1. How did you feel your appointment in the clinic went today?
2. Tell me about what happened during the appointment from your perspective…

- Prompts: Initial discussion; eye tests; outcomes.

1. Did you understand what the doctor/optometrist/nurse was telling you?
   1. When the doctor was mentioning XXXXX did that all make sense?
   2. Was there anything you would have liked to ask the doctor that you didn’t?
   3. Could anything have been made clearer for you?
2. You said XXXXX during the appointment. What were you trying to find out?
   1. Why was that important to you?
   2. Do you feel you had this issue resolved?
3. Can you think of anything else about the appointment you would like to have changed?
   1. If so, how would this have improved the appointment?

Prompt: access, organisation, staffing, frequency of appts

1. How did you feel about your eye condition at the end of the appointment?

**Appendix C2: Practitioner Semi-structured Interview Schedule**

This topic guide should be used as reference prior to conducting qualitative interviews with Health Care Practitioners (HCP) as part of the FENETRE Study’s Process Evaluation in the internal pilot and main study. The precise questions used will vary according to what was discussed and carried out during the Participant - Health Care Practitioners (HCP) consultation, with some questions referring directly to events within it. Examples of these types of question are included below with XXXXX marking content to be drawn from the observations taking place beforehand. The interviews will be semi-structured and will explore the perspectives of staff involved in routine nAMD hospital care and those who are participating in the community optometrist nAMD clinics.

**General Appointment Issues**

1. How did you feel your consultations with Participant went in the clinic today?
2. Tell me about what happened during the appointment of XXXX from your perspective…

- Prompts: Initial discussion; eye tests; outcomes.

1. You said XXXXX during the appointment. What were you trying to find out?
   1. Why was that important to you?
   2. Do you feel you had this issue resolved?
2. Can you think of anything else about the care provided for Participant with nAMD you would like to have changed?
   1. If so, how would this have improved the care?

Prompt: access, organisation, staffing, frequency of appts, resourses, training

1. How did you feel about the care at the end of the appointment?
2. Can you think of any other issues that could improve the care you gave today? If so, how would you able to overcome these issues?
