# Supplementary material for: Evaluation of training, patient and practitioner perspectives on community-based monitoring of patients with stable age-related macular degeneration compared to hospital-based care: The FENETRE study report no. 1
Source: Ophthalmic Physiol Opt. 2021 May 25;41(4):864–73. doi: 10.1111/opo.12836 (PMC12852252; doi:10.1111/opo.12836)
Supplement: Supplementary file 4 — Appendix S4. Qualitative analytical framework. [file 44402_2021_4104021_MOESM4_ESM.docx]

# *The FENETRE Study Report No. 1*

# Supplementary Appendix S4 – Qualitative Analytical Framework

| **REF** | **Theme / Node** |
| --- | --- |
| 001 | Variations in Practice |
| 001a | Between community sites |
| 001b | Between hospital sites |
| 001c | Between hospital and community sites |
| 001d | One site over time |
| 002 | Patient Acceptability |
| 002a | Community care |
| 002b | Hospital care |
| 003 | Practitioner Acceptability |
| 003a | Community optometrists |
| 003b | Hospital-based practitioners |
| 003c | Chains and Independents |
| 004 | Busyness of setting |
| 005 | Appointment structure |
| 005a | Clinical organisation |
| 005b | Follow-up period |
| 005c | Appointment length |
| 005d | Virtual clinics |
| 006 | Staffing |
| 007 | Technology |
| 008 | Patient-practitioner interactions |
| 009 | Clinical procedure issues |
| 010 | Patient outcomes |
| 011 | Mobility issues |
| 012 | Physical environment issues |
| 013 | Patient understanding of appointment / condition |
| 014 | Other patient sight or health problems |
| 015 | Transportation |
| 016 | FENETRE training |
| 017 | FENETRE purpose |
| 018 | Alternative pathways / suggestions |
| 019 | Patient referral / history |
